# Influence of Drying Methods on the Phenolic Content and Antioxidant Properties of *Crocus sativus* L. Tepals and Leaves By‐Products From Meknes (Morocco)

**DOI:** 10.1002/cbdv.202402967

**Published:** 2025-03-29

**Authors:** Soukaina Abou‐Wakil, Francesco Cacciola, Fatima Housti, Mohamed Rochd, Roberto Laganà Vinci, Luigi Mondello, Federica Davì, Nicola Micale, Natalizia Miceli, Maria Fernanda Taviano

**Affiliations:** ^1^ Faculty of Sciences, Department of Biology Moulay Ismail University Meknes Morocco; ^2^ Messina Institute of Technology c/o Department of Chemical, Biological, Pharmaceutical and Environmental Sciences, former Veterinary School University of Messina Messina Italy; ^3^ Chromaleont s.r.l., c/o Messina Institute of Technology c/o Department of Chemical, Biological, Pharmaceutical and Environmental Sciences, former Veterinary School University of Messina Messina Italy; ^4^ Department of Chemical, Biological, Pharmaceutical and Environmental Sciences University of Messina Messina Italy; ^5^ Foundation “Prof. Antonio Imbesi” University of Messina Messina Italy

**Keywords:** antioxidant activity, *Artemia salina* Leach, by‐products, drying method, phenolics, saffron

## Abstract

In recent years, much research has focused on the valorization of *Crocus sativus* by‐products as sources of bioactive compounds. This study was designed to investigate the impact of two different drying methods, freeze‐drying and air‐drying, on the phenolic content and antioxidant activity of leaves and tepals by‐products from *C. sativus* collected in Meknes, Morocco. Stigmas were included in the study to provide further information on this precious spice. The phenolic content of the hydroalcoholic extracts was determined spectrophotometrically and characterized by HPLC‐PDA/ESI‐MS analysis. The antioxidant properties were evaluated using different in vitro assays. Both in the DPPH and in the ferrous ion chelating activity assays, the extracts from freeze‐dried tepals (*Cs*‐TFD) and leaves (*Cs*‐LFD) displayed higher activity than those from air‐dried plant materials. *Cs*‐LFD showed the best scavenging activity, while superimposable chelating activity was observed for *Cs*‐TFD and *Cs*‐LFD. Finally, no toxicity was observed in the *Artemia salina* lethality bioassay.

## Graphical abstract

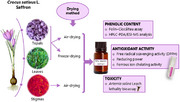

## 1 Introduction

*Crocus sativus* L. is an herbaceous perennial plant belonging to Iridaceae family. The corm is a subsoil organ providing vegetative propagation; it contains two or three apical buds and four to seven secondary buds. The apical buds sprout to produce leaves, a floral axis, and two or three daughter corms. The secondary buds produce a cauline axis and a clump of leaves [1]. The leaves develop from September to May, after which they begin to dry, corresponding to the beginning of the dormancy phase of the corms [2]. *C. sativus* has a subhysteranthous behavior, which means that the flowers can appear before, simultaneously, or after the leaves [1]. The flowers appear from October to the middle of November. They are composed of six violet tepals, three stamens, and a pistil, which contains an inferior ovary and a style ending with a red stigma divided into three filaments [3].

The name saffron is commonly applied to refer to both the plant and the dried stigmas, which represent the most precious spice in the world. The production of saffron spice generates enormous quantities of by‐products without any industrial application, which must thereby be discarded. It is estimated that saffron tepals and anthers represent about 90% of the whole flower's fresh weight; to yield 1 kg of stigmas, 63 kg of floral by‐products, and 1500 kg of foliage are wasted, as well as hundreds of bulbs that are too small and/or have morphological or biological alterations [4, 5, 6]. How to reuse these unexploited by‐products has become a major issue for farmers and industries. In fact, from the perspective of the circular economy, the sustainable use of agrifood wastes and/or by‐products to produce value‐added products for potential applications in the cosmetic, pharmaceutical, or food industries represents a crucial goal. In the last decades, many researchers have focused their attention on the valorization of *C. sativus* by‐products [1].

Numerous studies have demonstrated diverse pharmacological effects of *C. sativus*, including antioxidant, anticancer, antidiabetic, antineurodegenerative, anti‐inflammatory, and antidepressant [7, 8, 9]. The bioactive potential of this species was attributed to several secondary metabolites belonging to the phytochemical classes of carotenoids and polyphenols. Stigmas are mainly rich in apocarotenoids such as crocin, crocetin, safranal, and picrocrocin. Tepals contain flavonoids, including different classes such as flavones, flavonols, and flavanones, anthocyanins, and lutein diesters [10, 11]. Leaves are studied less compared to other plant parts. Their phytochemical composition comprises xanthones, flavonoids, isoflavonoids, and hydroxycinnamic acids [12].

Obtaining bioactive chemical components from plant sources depends on several factors, including the geographical location and the choice of the phenological stage of the plant, as well as the post‐harvest processes, mainly the methods applied for drying plant material, the extraction technique and the solvent [13, 14, 15, 16, 17].

A survey of recent literature revealed that saffron and *C. sativus* by‐products from some Moroccan regions such as Talioune, Oujda, and Boulmane have previously been investigated [18, 19, 20, 21, 22].

The present study was designed to evaluate the impact of the drying method on the phenolic content and the antioxidant activity of leaves and tepals by‐products from *C. sativus* collected in the locality of Ait Ouallal, Meknes, Morocco. The influence of drying methods on the phytochemical composition of *C. sativus* by‐products has been little investigated. Actually, few bibliographic data report studies on the effect of drying temperatures and air flows on the phenolic profile of floral bio‐residues composed by tepals, stamens, and styles; besides, research was carried out to compare the effect of different drying processes on the stability of anthocyanin extracts from tepals [6, 23, 24]. To our knowledge, similar studies have not been conducted previously on the leaves.

In this work, *C. sativus* leaves and tepals were subjected to two drying methods, namely freeze‐drying (FD) or lyophilization and air‐drying (AD); then, hydroalcoholic extracts were obtained from the plant material. The qualitative‐quantitative phenolic profile of *C. sativus* extracts was characterized by high‐performance liquid chromatography coupled to a photodiode array and electrospray ionization mass spectrometry (HPLC‐PDA/ESI‐MS) analysis. The antioxidant properties of the extracts were evaluated by different in vitro systems based on diverse mechanisms. In addition, the potential toxicity of *C. sativus* extracts was assessed by the *Artemia salina* lethality bioassay. Taking into account that the phytochemistry and biological potential of *C. sativus* from the Meknes region has not previously been investigated, it seemed interesting to extend the study to the stigmas as well.

## 2 Results and Discussion

### 2.1 Extraction Yields

The yields of *C. sativus* tepal, leaf, and stigma 70% MeOH extracts are reported in Table 1. The results showed that the yields varied depending on the plant part; higher extraction yields were found for stigma and tepal extracts, while the lowest yields were observed for leaf extracts.

**TABLE 1:** Yields of *Crocus sativus* tepal, leaf, and stigma hydroalcoholic extracts.

Plant material	Extract	Yield %
Tepals freeze‐dried	*Cs*‐TFD	46.75
Tepals air‐dried	*Cs*‐TAD	59.11
Leaves freeze‐dried	*Cs*‐LFD	28.05
Leaves air‐dried	*Cs*‐LAD	20.23
Stigmas air‐dried	*Cs*‐SAD	60.10

*Note*: The yields are referred to 100 g of dried plant material (dw).

Similarly to our results, in the work of Lahmass et al. carried out on ethanol extracts from different parts of *C. sativus* obtained by maceration for 24 h, stigma extract presented higher yield compared to leaf extract; nevertheless, these were far lower than those found in our study (28.76% and 4.3%) [20]. Our results agree with those previously reported by Ouahhoud et al. for extracts from *C. sativus* obtained by maceration for 24 h, three times, with 80% ethanol, showing higher extraction yields for tepals (69.45%) and stigma (64.57%) compared to that of leaf extract (31.19%) [18]. The results also agree with those of Benkerroum et al. for *C. sativus* tepal and stigma extracts obtained by maceration with 80% MeOH twice (45%–64% and 49.4%–66%, respectively) [25].

The comparison between the two drying methods highlighted different results for tepal and leaf extracts; in fact, for the tepal extracts, the yield of *Cs*‐TFD was slightly lower than that of *Cs*‐TAD, while for the leaf extracts *Cs*‐LFD and *Cs*‐LAD an inverse trend was observed.

### 2.2 Phytochemical Investigations

#### 2.2.1 Determination of Total Phenolic Content

The total phenolic content (TPC) of tepal, leaf, and stigma hydroalcoholic extracts of *C. sativus* is reported in Table 2. The results highlighted significant differences between the TPC values in the different plant parts. Both the tepal extracts exhibited the highest phenolic content (65.94 ± 1.15 mg gallic acid equivalents [GAE]/g extract for *Cs*‐TFD and 57.01 ± 1.59 mg GAE/g extract for *Cs*‐TAD) compared to leaf and stigma extracts, which instead showed a quite similar quantity of total phenolics. The TPC of the extracts decreases in the following order: *Cs*‐TFD > *Cs*‐TAD > *Cs*‐SAD > *Cs*‐LFD > *Cs*‐LAD.

**TABLE 2:** Total phenolic content of *Crocus sativus* tepal, leaf, and stigma hydroalcoholic extracts.

*Crocus sativus* extract	TPC (mg GAE/g extract)
*Cs*‐TFD	65.94 ± 1.15^a^
*Cs*‐TAD	57.01 ± 1.59^b^
*Cs*‐LFD	41.26 ± 2.74^c^
*Cs*‐LAD	37.90 ± 0.57^d^
*Cs*‐SAD	42.88 ± 1.66^c^

*Note*: Values are expressed as the mean ± SD. Different superscript letters (a–d) indicate significant differences at *p* < 0.05 by Duncan's test.

From the comparison of TPC values and the yields obtained for the extracts, it was highlighted that the stigma extract, exhibiting an extraction yield close to that of tepal extracts, showed a phenolic content comparable to that of the leaf extracts, whose extraction yields were approximately half and a third. Similar findings have been previously reported in other works [18, 20].

Among the extracts obtained from *C. sativus* by‐products subjected to the two drying methods, a correspondence between extraction yields and TPC was observed only for leaf extracts, while for tepal extracts, greater quantities of phenolic compounds were extracted in *Cs*‐TFD, exhibiting a lower yield than *Cs*‐TAD.

Notably, the results of our investigations indicate that the drying method significantly affects the TPC of the plant material, FD being more effective than AD. Drying process conditions, such as temperature, air flow, and duration, can allow stability or alter the TPC of the plant material. Phenolic compounds might be degraded either by high temperature or by oxidation; therefore, a considerable amount of polyphenols might be lost during the drying process [6]. The favorable effect of the FD process is associated with the elimination of water from the plant material by sublimation rather than a long stay at room temperature or the use of high temperatures. This allows for less alteration of the physicochemical quality and the preservation of the active compounds [26].

The TPC highlighted in our extracts was found to be far higher than those reported in previous investigations. Lahmass et al. determined the TPC of ethanol extracts obtained by maceration at room temperature for 24 h from different air‐dried parts of *C. sativus* harvested in Oujda, located in the north‐east of Morocco, resulting in 23.32 µg GAE/mg extract 16.63 µg GAE/mg extract for leaf and stigma extract, respectively [20]. In another study, the TPC of ethanol extracts obtained from air‐dried leaves and stigmas of Indian *C. sativus* were 5.62 and 8.28 mg/g, respectively [27]. On the other hand, the amount of TPC of the MeOH extract obtained by maceration at 35°C for 24 h of dried tepals from *C. sativus* collected in Taliouine, Morocco, was 65.34 mg GAE/g extract, superimposable to our values [19]. Similar TPC values were also found by Ouahhoud et al. in 80% ethanol extracts obtained by maceration for 24 h, three times, of oven dried (37°C) tepals, leaves, and stigmas from *C. sativus* cultivated in Taliouine (64.66 µg GA eq/mg extract, 38.56 µg GA eq/mg extract, and 34.41 µg GA eq/mg extract, respectively) [18]. In a recent work published by Benkerroum et al., tepals and stigmas from *C. sativus* cultivated in Taliouine shade‐dried at room temperature and extracted by maceration with 80% MeOH contained phenolic compounds at the rate of 64.73 and 56.11 mg GAE/g extract [25].

It should be underlined that the comparison of our results with previous studies highlighted that the application of ultrasound at 50°C allowed efficient phenolic extraction in much shorter times than maceration.

#### 2.2.2 Phenolic Characterization by HPLC‐PDA/ESI‐MS Analysis

**FIGURE 1:**
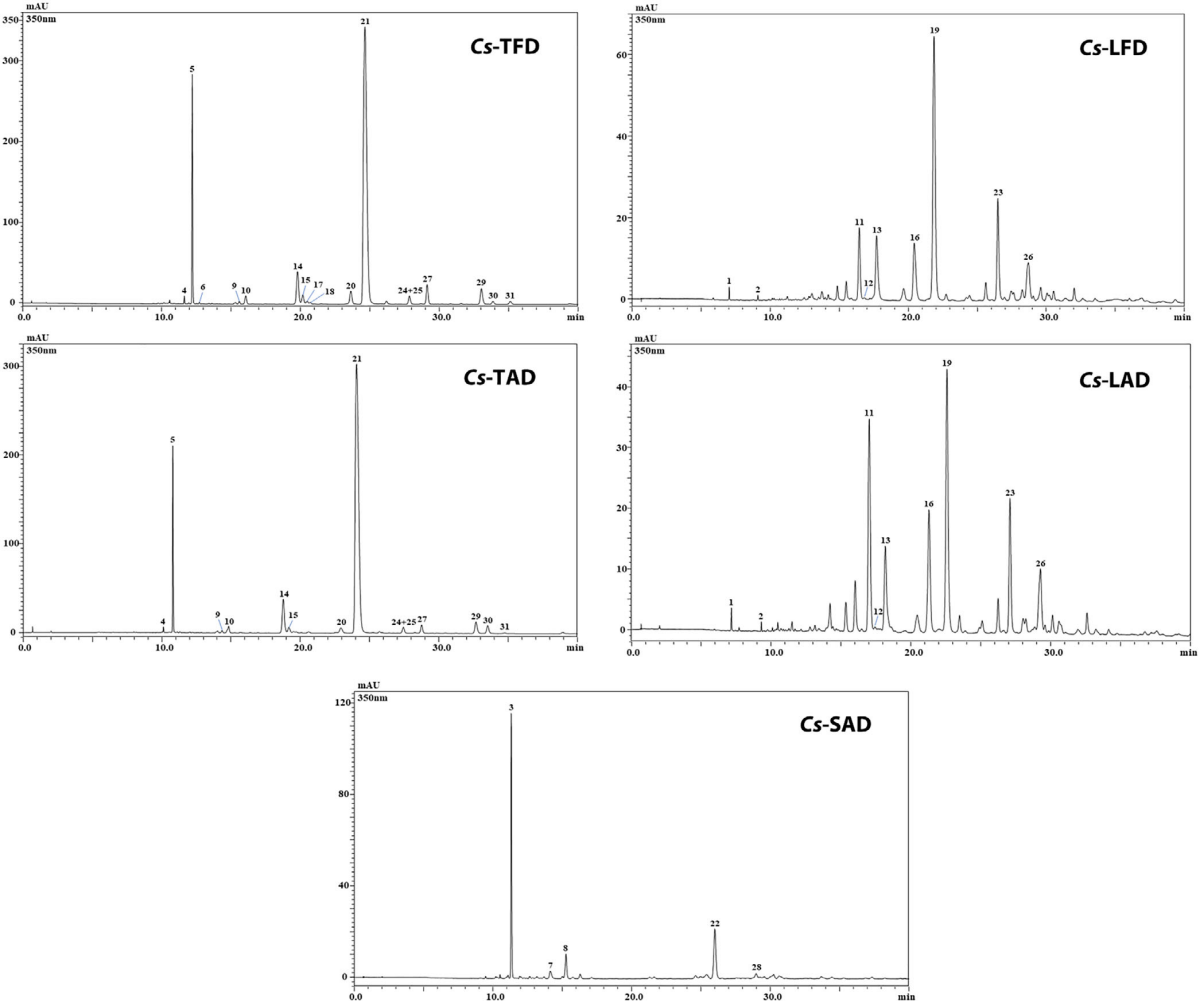
HPLC‐PDA analysis of *Crocus sativus* tepal, leaf, and stigma hydroalcoholic extracts (chromatograms extracted at 350 nm). Tentative identification is shown in Table 3.

Phenolic profile analyses were carried out using HPLC‐PDA/ESI‐MS (Figure 1). As listed in Table 3, a total of 31 phenolic compounds were detected according to *λ*_max_, retention times, mass spectrometry, and literature data. Compounds were mainly assigned to flavonoids, such as kaempferol, quercetin, and isorhamnetin derivatives, with different isomers present among tepals, leaves, and stigmas; while leaf extracts were the only ones in which the presence of different acetophenone derivatives was highlighted.

**TABLE 3:** Semi‐quantification of phenolic compounds in *Crocus sativus* tepal, leaf, and stigma hydroalcoholic extracts through LC‐PDA/ESI‐MS analysis.

Peak	Compound	*t*_R_ (min)	UV max (nm)	[M−H]^−^	*Cs*‐TFD	*Cs*‐TAD	*Cs*‐LFD	*Cs*‐LAD	*Cs*‐SAD	Ref.
1	Trihydroxy‐methoxyacetophenone‐diglucoside	7.03	286, 335sh	545^a^, 521, 197	–—	—	x×	×	—	—
2	Unknown	9.11	287, 335sh	631^a^, 607, 197	—	—	×	×	—	—
3	Kaempferol triglucoside	11.33	265, 346	771, 609, 447, 285	—	—	—	—	5.02 ± 0.039	[28]
4	Myricetin diglucosyl‐rhamnoside	11.65	254, 351	787, 625, 463	×	×	—	—	—	[22]
5	Kaempferol triglucoside	12.23	265, 346	771, 609, 285	11.65 ± 0.003	7.39 ± 0.012	—	—	—	[29]
6	Kaempferol rutinosyl‐hexoside	12.74	264, 347	755	×	—	—	—	—	[22]
7	Unknown	14.14	250	375, 167	—	—	—	—	×	—
8	Kaempferol triglucoside	15.28	265, 341	771, 609, 447, 285	—	—	—	—	0.66 ± 0.007	[28]
9	Kaempferol glucosyl‐(6″‐acetylgalactoside)‐hexoside	15.62	265, 348	813, 651	×	×	—	—	—	[22]
10	Kaempferol sophoroside	16.08	264, 344	609, 447	0.81 ± 0.001	0.57 ± 0.003	—	—	—	[22]
11	Quercetin triglucoside	16.45	272, 334	787, 625, 463, 301	—	—	1.68 ± 0.033	4.20 ± 0.040	—	—
12	4′,6′‐Dihydroxy‐2′‐methoxyacetophenone 6′‐glucoside	17.23	284, 320sh	367^a^, 343, 181	—	—	×	×	—	[30]
13	2,3,4‐Trihydroxy‐6‐methoxyacetophenone‐3‐β‐d‐glucopyranoside	17.71	285, 329sh	383^a^, 359, 197	—	—	×	×	—	[30]
14	Quercetin sophoroside	19.81	255, 352	625, 463, 301	5.18 ± 0.018	4.74 ± 0.000	—	—	—	[22]
15	Quercetin sophoroside	20.19	264, 343	625, 463	1.18 ± 0.009	0.41 ± 0.002	—	—	—	[22]
16	Isorhamnetin sophorosyl‐rhamnoside	20.44	253, 351	785, 315	—	—	2.00 ± 0.004	2.74 ± 0.006	—	[28]
17	Kaempferol sophoroside	20.50	265, 339	609, 447	0.14 ± 0.003	—	—	—	—	[22]
18	Luteolin (or kaempferol) malonyl‐dihexoside	20.72	265, 347	695, 447	×	—	—	—	—	—
19	6‐Hydroxyluteolin 7‐glucoside	21.87	268, 349	447	—	—	5.68 ± 0.007	3.43 ± 0.007	—	[31]
20	Isorhamnetin sophoroside	23.66	265, 343	639, 477, 315	2.11 ± 0.002	0.54 ± 0.007	—	—	—	[22]
21	Kaempferol sophoroside	24.67	265, 346	609, 447, 285	71.44 ± 0.060	63.84 ± 0.113	—	—	—	[22]
22	Kaempferol sophoroside	26.02	265, 347	609, 447, 285	—	—	—	—	2.57 ± 0.007	[28]
23	Kaempferol 3,7,4′‐triglucoside	26.50	271, 335	771	—	—	2.64 ± 0.011	2.26 ± 0.005	—	[29]
24 + 25	Kaempferol rutinoside + quercetin hexoside	27.87	264, 347	593, 463, 285 + 463, 301	0.97 ± 0.016	0.54 ± 0.008	—	—	—	[22]
26	Trihydroxy‐methoxyacetophenone‐acetylglucopyranoside	28.65	285, 329sh	401, 359, 197	—	—	×	×	—	—
27	Isorhamnetin rutinoside	29.14	253, 353	623, 477, 315	2.48 ± 0.019	0.76 ± 0.000	—	—	—	[22]
28	Vanillic acid	29.29	287	169^b^	—	—	—	—	×	[32]
29	Kaempferol glucoside	33.05	265, 346	447, 285	2.53 ± 0.009	1.61 ± 0.008	—	—	—	[22]
30	Kaempferol‐(6″‐acetyl‐glucoside)‐glucoside	33.88	266, 347	651, 489, 285	0.17 ± 0.005	0.83 ± 0.009	—	—	—	[22]
31	Isorhamnetin glucoside	35.13	267, 347	477, 315	0.25 ± 0.007	×	—	—	—	[22]

*Note*: Quantification of phenolic compounds is reported in mg/g of dried extract ± SD (*n* = 3). ×: detected, but not quantified; sh: wavelength shoulder.

^a^ Detected as [M+Na]^+^.

^b^ Detected as [M+H]^+^.

In general, among the understudied extracts, tepal ones were the quali‐quantitatively richest, especially in terms of kaempferol derivatives. In particular, a total of 16 compounds were detected in *Cs*‐TFD and 13 in *Cs*‐TAD; for almost all compounds detected in tepal extracts, a greater amount of these was found in *Cs*‐TFD. Among the phenolic compounds identified, the kaempferol‐sophoroside isomer turned out to be the most abundant one in both tepal extracts (71.44 ± 0.060 and 63.84 ± 0.113 mg/g, for *Cs*‐TFD and *Cs*‐TAD, respectively), followed by kaempferol triglucoside (11.65 ± 0.003 and 7.39 ± 0.012 mg/g, for *Cs*‐TFD and *Cs*‐TAD, respectively).

In both leaf extracts, *Cs*‐LFD and *Cs*‐LAD, a superimposable qualitative profile was highlighted, with a total of nine compounds detected. Differently from tepal extracts, 6‐hydroxyluteolin 7‐glucoside was the most abundant compound in *Cs*‐LFD extract (5.68 ± 0.007 mg/g), while in *Cs*‐LAD was quercetin triglucoside (4.20 ± 0.040 mg/g).

Finally, the analysis of stigma extract *Cs*‐SAD led to the identification of five phenolic compounds, kaempferol triglucoside being the main component detected in the extract (5.02 ± 0.039 mg/g).

### 2.3 *A. salina* Leach Lethality Bioassay

The assessment of toxicity in medicinal plant extracts plays a crucial role in ensuring their safety and efficacy. The microcrustacean *A. salina* is widely used as a model organism for the preliminary estimation of the toxicity of plant extracts, as it offers a number of significant advantages such as ease of cultivation, cost‐effectiveness, rapid response, and reproducibility [33]. Within this perspective, the *A. salina* lethality bioassay was used for toxicity assessment of *C. sativus* tepal, leaf, and stigma extracts. Based on Clarkson's toxicity criterion applied for the assessment of the degree of toxicity, the results obtained after 24 h of incubation showed no toxicity against brine shrimp larvae for all the extracts, with LC_50_ values over 1000 µg/mL [34].

### 2.4 Antioxidant Activity

Antioxidants are considered health‐supporting compounds due to their ability to protect biological systems from oxidative damage produced by reactive oxygen species (ROS). Plant‐derived antioxidants have received a great deal of attention because they play a pivotal role in the prevention and/or treatment of several disorders related to oxidative stress, such as cardiovascular, inflammatory, and neurodegenerative diseases, diabetes, cancers, as well as aging [5, 35].

As is known, various methods based on different mechanisms should be used to estimate the antioxidant properties of plant extracts since numerous activities are involved in the process of oxidation inhibition by antioxidant compounds [36]. Antioxidants have been traditionally divided into two classes: primary or chain‐breaking antioxidants (mainly acting by ROS/RNS scavenging) and secondary or preventative antioxidants (usually acting by transition metal ion chelation) [37]. Depending on the mechanism of the chemical reactions involved, antioxidant assays are based on electron transfer (ET), hydrogen atom transfer (HAT), or chelation of transition metals. The ET mechanism involves a redox reaction with an oxidant as an indicator of the reaction endpoint. HAT‐based methods are generally composed of a synthetic free radical generator, an oxidizable molecular probe, and an antioxidant [38]. In addition to ET and HAT mechanisms, estimating the antioxidant capacity of an extract or compound of metal ion chelation can also be considered one of the most important mechanisms of action of secondary antioxidants. Metal chelators reduce the amount of available transition metals, thus decreasing the extent of hydroxyl radicals generated by the Fenton reaction and limiting metal ion‐induced lipid oxidation [36]. Recent investigations have demonstrated that transition metals such as Fe^2+^ and Cu^2+^ are responsible for the pathogenesis of several diseases, including neurodegenerative (Alzheimer's and Parkinson's) and cardiovascular diseases [38].

Among the most important groups of plant‐based antioxidants are phenolic compounds, which have one or more aromatic rings with one or more hydroxy groups; these compounds have attracted increasing attention due to their powerful antioxidant properties and their beneficial effects in the prevention of various oxidative stress‐related diseases [39]. Phenolic compounds can act as hydrogen or electron donors, capable of stabilizing unpaired electrons (radicals) and chelating transition metal ions, resulting from different conjugations and varying numbers of hydroxyl groups [40].

In the present work, to determine and compare the antioxidant efficacy of the hydroalcoholic extracts of *C. sativus* tepals and leaves by‐products subjected to different drying procedures, along with stigma extract, three in vitro tests were performed, namely the 2,2‐diphenyl‐1‐picrylhydrazyl (DPPH) and the reducing power assays to evaluate the primary antioxidant properties, and the ferrous ion chelating activity assay to assess the secondary antioxidant properties.

Regarding the DPPH test, the free radical scavenging activity of the extracts was determined by the capacity to reduce the stable free radical DPPH• to the non‐radical form DPPH‐H. This reduction, based on the dual mechanism of HAT and ET, is indicated by the color change of the solution from purple to yellow. The degree of discoloration shows the efficiency of the antioxidant activity of plant extracts [27].

**FIGURE 2:**
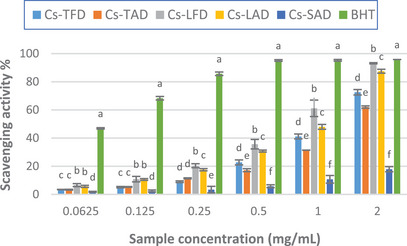
DPPH radical scavenging activity (%) of *Crocus sativus* tepal, leaf, and stigma hydroalcoholic extracts. The results are expressed as the mean ± SD (*n* = 3).

The results of the DPPH test showed that the extracts exhibited radical scavenging activity in the range of concentrations tested (0.0625–2 mg/mL), which augments with the increase of extract concentration. As depicted in Figure 2, both the leaf extracts exhibited higher activity than those from tepals and stigmas, the latter displaying moderate scavenging activity only, as compared to the standard BHT. At the highest concentration tested, the activity of the extracts ranged between 17.9% (*Cs*‐SAD) and 93.2% (*Cs*‐LFD), the latter close to that of standard BHT (95.8%). Furthermore, the extracts obtained from freeze‐dried plant material, namely *Cs*‐TFD and *Cs*‐LFD, were shown to display greater scavenging activity compared to those from air‐dried plant material *Cs*‐TAD and *Cs*‐LAD. The IC_50_ values calculated for *C. sativus* extracts confirmed these results, demonstrating the best activity for *Cs*‐LFD (0.93 ± 0.05 mg/mL), and the lowest activity for *Cs*‐SAD (IC_50_ > 2 mg/mL) (Table 4).

**TABLE 4:** DPPH radical scavenging activity, reducing power, and ferrous ion chelating activity of *Crocus sativus* tepal, leaf, and stigma hydroalcoholic extracts.

*Crocus sativus* extract	DPPH scavenging activity, IC_50_ (mg/mL)	Reducing power (ASE/mL)	Chelating activity, IC_50_ (mg/mL)
*Cs*‐TFD	1.34 ± 0.05^d^	18.17 ± 2.45^c^	0.42 ± 0.02^b^
*Cs*‐TAD	1.60 ± 0.01^e^	9.22 ± 0.52^b^	0.88 ± 0.19^c^
*Cs*‐LFD	0.93 ± 0.05^b^	12.27 ± 3.92^b^	0.44 ± 0.04^b^
*Cs*‐LAD	1.06 ± 0.01^c^	12.04 ± 1.05^b^	0.81 ± 0.06^c^
*Cs*‐SAD	ND	12.15 ± 2.84^b^	0.92 ± 0.06^c^
Standard	BHT	BHT	EDTA
	0.06 ± 0.01^a^	1.44 ± 0.02^a^	0.02 ± 6.86E−05^a^

*Note*: The results are expressed as the mean ± SD (*n* = 3). Different superscript letters (a–e) within the same column indicate significant differences at *p* < 0.05 by Duncan's test.

Our results agree with those previously reported in other studies, which showed good free radical scavenging activity for *C. sativus* leaf and tepal extracts and weak activity for the stigma extract [5, 20, 25]. Differently, in the study reported by Baba et al. stigma extracts exhibited the highest activity, whereas the leaf extracts resulted in much less active [27].

As demonstrated by both spectrophotometric determination and HPLC‐PDA/ESI‐MS analysis, *C. sativus* tepal extracts, *Cs*‐TFD and *Cs*‐TAD, are richer in phenolic compounds, mainly flavonoids such as kaempferol, quercetin, and isorhamnetin derivatives. Nonetheless, the best radical scavenging activity was observed for the leaf extracts; this could be explained by considering their phenolic profile, which was different, both qualitatively and qualitatively. Among the phenolics identified in *Cs*‐LFD, 6‐hydroxyluteolin 7‐glucoside resulted in the most abundant; it was found in greater amount than *Cs*‐LAD, while it was not detected in the other extracts. The good free radical scavenging properties of 6‐hydroxyluteolin 7‐glucoside have been previously reported; the presence of this compound could explain, almost in part, the observed results [41]. Furthermore, among detected phenolic compounds, the presence of four acetophenone derivatives was highlighted exclusively in *Cs*‐LFD and *Cs*‐LAD; nonetheless, previous studies have shown moderate to mild DPPH free radical scavenging properties for several acetophenone glycosides [42, 43].

The reducing power of *C. sativus* extracts was evaluated by the potassium ferricyanide method, an ET‐based assay measuring the reduction of Fe(III) to Fe(II). Compounds with reduction potential react with potassium ferricyanide (Fe^3+^) to form potassium ferrocyanide (Fe^2+^), which subsequently reacts with ferric chloride to form a ferric–ferrous complex. The color of the solution changes from yellow to different shades of green and blue, having an absorption maximum of 700 nm [44]. An increase in absorbance is proportional to an increase in the reducing power [45, 46].

**FIGURE 3:**
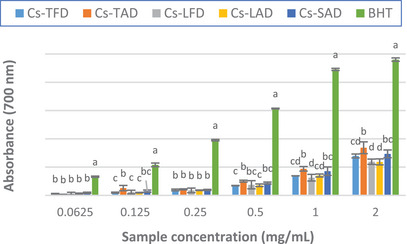
Reducing power (Abs 700 nm) of *Crocus sativus* tepal, leaf, and stigma hydroalcoholic extracts. The results are expressed as the mean ± SD (*n* = 3).

The results of the assay, depicted in Figure 3 and Table 4, showed that all the extracts displayed moderate reducing power as compared to the standard. Among the extracts, *Cs*‐TAD exhibited the best activity, slightly higher than that of *Cs*‐TFD, with an absorbance value of 0.84 ± 0.10 at the maximum tested concentration (2 mg/mL), approximately three times lower than that of the standard BHT (2.40 ± 0.10), and an ASE/mL value of 9.22 ± 0.52.

In agreement with our results, in the study of Baba et al. moderate reducing power was observed for all the *C. sativus* extracts tested, greater for the stigma extracts than the leaves ones [27]. Our results disagree with those previously reported by Lahmass et al., who showed good reducing power for *C. sativus* stigma extracts, much greater than those of the leaves [20].

The chelating properties of the extracts were assessed by determining the extent of Fe^2+^–ferrozine complex formation. Chelation of this metal ion with ferrozine leads to the formation of a red‐colored complex; in the presence of chelating agents, the formation of the complex is stopped, and the red color decreases. Measuring the rate of color reduction allows the estimation of the chelating activity [5].

**FIGURE 4:**
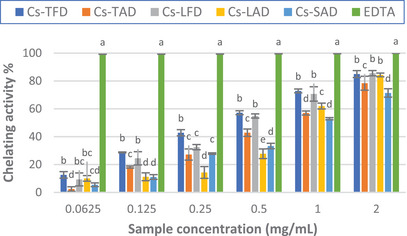
Ferrous ion chelating activity (%) of *Crocus sativus* tepal, leaf, and stigma hydroalcoholic extracts. The results are expressed as the mean ± SD (*n* = 3).

The results of the assay showed that the extracts exhibited good chelating properties compared to the standard EDTA. As depicted in Figure 4, for almost all concentrations tested, a significant difference was observed between the two diverse drying methods; indeed, the activity of the extracts from freeze‐dried tepals and leaves was higher than those from air‐dried plant material, reaching approximately 85% for both extracts at the highest concentration tested. The IC_50_ values calculated for the extracts also confirmed these results; in fact, *Cs*‐TFD and *Cs*‐LFD showed the lowest IC_50_, which resulted in superimposable (0.42 ± 0.02 and 0.44 ± 0.04 mg/mL, respectively) and about half that of *Cs*‐TAD and *Cs*‐LAD (Table 4).

The stigma extract *Cs*‐SAD was found to display the lowest activity, reaching approximately 71% at the highest concentration tested; nonetheless, the IC_50_ calculated for the extract was close to that of *Cs*‐TAD and *Cs*‐LAD.

The obtained results do not agree with those previously reported by Ouahhoud et al., who found the best ferrous ion chelating activity for 80% ethanol extract of *C. sativus* tepals, followed by that of the stigmas, whereas the leaf extract resulted in the less active [18]. Our results also differ from those of Sánchez‐Vioque et al., who found low ferrous ion chelating activity for *C. sativus* leaf extract and negligible activity for the tepal extract [5].

Despite the different TPC, as well as the qualitative–quantitative phenolic profiles, both *Cs*‐TFD and *Cs* LFD showed comparable chelating activity, which was higher than that of the other extracts. Many studies have confirmed that flavonoids can behave as antioxidants because of their chelating properties [47]. The presence of 6‐hydroxyluteolin 7‐glucoside could partly explain the good chelating properties of *Cs*‐LFD; indeed, it has been previously demonstrated that 6‐hydroxyluteolin is an effective chelator of Fe^2+^ ions [48].

Overall, the results of antioxidant assays showed that the extracts from *C. sativus* tepals and leaves by‐products harvested from the Meknes region could be exploited as natural sources of phenolic antioxidants displaying good radical scavenging and Fe^2+^ chelating properties and that FD improves the recovery of antioxidant compounds, which are partially lost with the AD process. Taking into account the better antioxidant activity displayed by the leaf extracts, which showed a lower phenolic content than tepal ones, it cannot be ruled out that phenolics are not the only ones responsible for the highlighted antioxidant properties and that other polar antioxidant compounds belonging to different chemical classes may be contained in the phytocomplexes, such as carotenoids, present in both leaves and tepals of *C. sativus*, which are well‐known to be effective antioxidants, in particular, xanthophylls such as lutein, which are more polar than carotenes [11, 25, 49]. Polysaccharides, previously detected in *C. sativus* leaves and tepals, may also contribute to the observed activity [12, 50, 51].

The results of our investigations add information to previous studies on the influence of drying methods on the phenolic recovery from *C. sativus* floral by‐products and provide information on this topic relating to leaves for the first time [6, 23, 24]. These findings further support the studies aimed at the valorization of *C. sativus* by‐products, indicating freeze‐drying as a strategy to increase the production of natural antioxidants, which can be considered as active ingredients in food supplements, functional foods, and beverages in pharmaceutical preparations and cosmetic formulations. This could further improve the use and exploitation of these by‐products, becoming a valuable source of income while reducing biowaste, thus increasing the profitability and sustainability of saffron production.

## 3 Conclusions

In this work, the influence of the drying method on the phenolic content and the antioxidant activity of leaves and tepals by‐products from *C. sativus* collected in the locality of Ait Ouallal, Meknes, Morocco, was investigated. Since the plant growing in this area had not previously been studied, it seemed interesting to include the stigmas in our research, to provide additional information on this commercially precious spice. The comparative study carried out on the hydroalcoholic extracts of *C. sativus* tepals and leaves clearly showed that the use of FD allows the preserve higher quantities of antioxidant compounds, which are instead partly lost with the AD process. Overall, the results of the different antioxidant assays highlighted both primary and secondary antioxidant activity for the extracts, which, therefore, could be exploited as a valuable source of antiradical and iron‐chelating compounds. The good chelating properties of the extracts from freeze‐dried plant material deserve particular interest. At last, the *A. salina* lethality bioassay showed the absence of toxicity for all the extracts, indicating their potential safety.

The results of the present study provide further insights into the knowledge of efficient strategies for the recovery of natural antioxidant biomolecules from *C. sativus* tepals and leaves by‐products that may be applied in the food, pharmaceutical, and cosmeceutical industries.

## 4 Experimental Section

### 4.1 Chemicals and Reagents

LC/MS‐grade water (H_2_O), acetonitrile (ACN), quercetin 3‐*O*‐rhamnoside, luteolin‐7‐*O*‐glucoside, Folin–Ciocâlteu reagent, sodium carbonate, gallic acid, DPPH, butylated hydroxytoluene (BHT), potassium hexacyanoferrate(III), iron(III) chloride hexahydrate, l‐ascorbic acid, sodium phosphate monobasic monohydrate, potassium phosphate dibasic, trichloroacetic acid, iron(II) chloride, 3‐(2‐pyridyl)‐5,6‐diphenyl‐1,2,4‐triazine‐4′,4″‐disulfonic acid sodium salt (ferrozine), and ethylenediaminetetraacetic acid (EDTA) disodium salt dihydrate, were obtained from Merck Life Science (Merck KGaA, Darmstadt, Germany). Methanol (MeOH) was purchased from Omniascientific snc (Messina, Italy).

### 4.2 Plant Material

The tepals and stigmas of *C. sativus* L. were harvested in November 2022; the leaves were collected in February 2023 in the locality of Meknes, specifically in Ait Ouallal region (33°50′28.536″ N–5°35′43.361″ W). The plant taxonomic identification was performed by Prof. Ouafae Benkhnigue at the Scientific Institute of Rabat, Morocco. A voucher specimen under the number RAB114620 has been deposited in the herbarium of the University Mohammed V, Rabat, Morocco.

### 4.3 Drying Procedures

For tepals and leaves two different methods were utilized, namely FD and AD. For FD process, tepals and leaves were immediately frozen after collection and successively freeze‐dried by using a lyophilizer model Alpha 1‐4 LD plus (Christ, Germany) at −53°C; the AD process was performed indoors at room temperature for 1 week. The stigmas were air‐dried indoors for 4 days.

### 4.4 Extraction Procedure

The dried plant material was ground; then, 1 g of each sample was subjected to preventive maceration for 1 h at 25°C using a MeOH–H_2_O mixture (70:30 v/v), with a sample‐to‐solvent ratio of 1:10 (w/v). The extraction was performed using 70% MeOH (plant material‐to‐solvent ratio of 1:10 w/v) in an ultrasonic bath at 50°C for 15 min; the procedure was repeated three times. After filtration, the extraction solutions were combined and centrifuged at 3000 rpm for 10 min at room temperature; then, the supernatants were evaporated to dryness by high‐performance solvent evaporation system EZ‐2 Plus (Genevac Ltd., Ipswich, UK). The yields of the extracts, referred to 100 g of dried plant material, are reported in Table 1.

### 4.5 Phytochemical Investigations

#### 4.5.1 Determination of TPC

The TPC of *C. sativus* tepal, leaf, and stigma hydroalcoholic extracts was estimated according to the colorimetric method described by Gao et al. [52]. Briefly, a volume of 0.1 mL of each sample solution was mixed with 0.2 mL of Folin–Ciocâlteu reagent; then, 2 mL of distilled water and 1 mL of 15% sodium carbonate were added. The mixture was kept for 2 h at room temperature in the dark; afterward, the absorbance was measured at 765 nm by UV‐1601 spectrophotometer (Shimadzu, Milan, Italy). For the quantitative estimation of total polyphenols, the calibration curve of gallic acid was used. The TPC was expressed as mg GAE/g of extract (dw) ± standard deviation (SD).

#### 4.5.2 Phenolic Profile Characterization by HPLC‐PDA/ESI‐MS Analysis

The tepal, leaf, and stigma hydroalcoholic extracts of *C. sativus* were analyzed by HPLC‐PDA/ESI‐MS to characterize their phenolic profile [22, 28–32].

*Sample preparation*: The dried extracts were redissolved in 70% MeOH (10 mg/mL). For the chromatographic separation, an injection volume of 2 µL was employed, and the analysis was performed in triplicate.

*HPLC/MS analytical condition*: Chromatographic analysis was accomplished by means of a Shimadzu HPLC system (Kyoto, Japan) equipped with a CBM‐20A controller, two LC‐20AD dual‐plunger parallel‐flow pumps, a DGU20A5R degasser, a CTO‐20AC column oven, a SIL‐30AC autosampler, an SPD‐M20A photodiode array detector, and an LCMS‐2020 single quadrupole mass spectrometer, with the employment of ESI source operated in negative and positive ionization modes. Chromatographic separations were carried out on Ascentis Express RP C18 columns (150 × 2.1 mm; 2.7 µm) (Merck Life Science, Merck KGaA, Darmstadt, Germany). The employed mobile phase was composed of two solvents: water (Solvent A) and ACN (Solvent B) both acidified with 0.1% of formic acid v/v. The flow rate was set at 0.5 mL/min, under gradient elution 0 min—0% B, 10 min—10% B, 20 min—11% B, 30 min—15% B, 50 min—18% B, 65 min—23% B, 70 min—100% B, 75 min—100% B. PDA was applied in the range of 190–400 nm and monitored at a wavelength of 350 nm (sampling frequency: 40, time constant: 0.080 s). MS conditions were as follows: scan range and the scan speed were set at a mass‐to‐charge ratio (*m*/*z*) 100–1600 and 7500 amu/s, respectively; event time: 0.3 s, nebulizing gas (N_2_) flow rate: 1.5 L/min, drying gas (N_2_) flow rate: 15 L/min, interface temperature: 350°C, heat block temperature: 300°C, DL temperature: 300°C, DL voltage: 1 V, interface voltage: M‐ 4.5 kV.

*Standards*: Calibration curves of two standards (quercetin 3‐*O*‐rhamnoside and luteolin‐7‐*O*‐glucoside) were employed for the quantification of the flavonoid content in sample extracts. Each analysis was performed in 6 repetitions. Data acquisition was performed by Shimadzu LabSolution software ver. 5.99. Quercetin 3‐*O*‐rhamnoside (0.1, 1, 10, 50, 100 ppm), *y* = 8054*x* + 27 465, *R* ^2^ = 0.9997, LOD = 0.034, LOQ = 0.103; luteolin‐7‐O‐glucoside (0.1, 1, 10, 50, 100 ppm), *y* = 12 881*x* + 54 935, *R* ^2^ = 0.9996, LOD = 0.016, LOQ = 0.048.

### 4.6 *A. Salina* Leach Lethality Bioassay

The toxicity of *C. sativus* tepal, leaf, and stigma hydroalcoholic extracts was investigated by performing the brine shrimp (*A. salina* Leach) lethality bioassay, according to the protocol previously reported by Meyer et al., with some modifications [53]. *A. salina* cysts were placed in a hatchery dish containing artificial seawater (32 g sea salt/L distilled water) and incubated for hatching under a 60 W lamp, at a temperature of 24°C–26°C. Twenty‐four hours after hatching, active nauplii free from eggshells were collected from the brighter portion of the hatchery dish. For the assay, ten brine shrimp larvae were placed in plates containing 5 mL of artificial seawater mixed with different amounts of the extracts appropriately dissolved to obtain final concentrations in the range 10–1000 µg/mL. After incubation at 24°C–26°C for 24 h, live larvae were counted; then, the median lethal concentration (LC_50_) values were estimated. Three replicates of each sample concentration were tested. To assess the toxicity level of the extracts, Clarkson's toxicity criterion was used [34].

### 4.7 Antioxidant Activity

#### 4.7.1 Free Radical Scavenging Activity

The DPPH assay was used to determine the free radical scavenging activity of *C. sativus* tepal, leaf, and stigma hydroalcoholic extracts, according to the method of Ohnishi et al. [54]. For each extract, concentrations ranging from 0.0625 to 2 mg/mL were prepared; a 0.5 mL aliquot of each sample solution was mixed with 3 mL of DPPH methanol solution (0.1 mM). After incubation of the mixture for 20 min in the dark at room temperature, the absorbance was read at 517 nm using a model UV‐1601 spectrophotometer (Shimadzu). For the control, 0.5 mL solvent was used instead of the sample solution. BHT was used as the reference standard. The results are expressed as scavenging activity (%). The mean 50% inhibitory concentration (IC_50_) values were also determined.

#### 4.7.2 Reducing Power

The evaluation of Fe^3+^–Fe^2+^ transformation by the potassium ferricyanide method was used to assess the reducing power of *C. sativus* extracts [55]. For each extract, concentrations ranging from 0.0625 to 2 mg/mL were prepared. A mixture containing 1 mL of each sample solution, 2.5 mL of phosphate buffer (0.2 M, pH 6.6), and 2.5 mL of 1% potassium ferricyanide was prepared and incubated for 20 min at 50°C. After rapid cooling, 2.5 mL of 10% trichloroacetic acid was added; then, the mixture was centrifuged at 3000 rpm for 10 min. A volume of 2.5 mL of the supernatant was taken and mixed with 2.5 mL of distilled water and 0.5 mL of 0.1% ferric chloride. After 10 min incubation at room temperature and in the dark, the absorbance of the samples was measured at 700 nm. BHT and ascorbic acid were used as reference standards. The results are expressed as mean absorbance values ± SD and ascorbic acid equivalent (ASE/mL) ± SD.

#### 4.7.3 Ferrous Ion (Fe^2+^) Chelating Activity

The Fe^2+^ chelating activity of *C. sativus* extracts was estimated according to the method reported by Kumar et al. [56]. For each extract, concentrations ranging from 0.0625 to 2 mg/mL were prepared. An aliquot of 1 mL of each sample solution was mixed with 0.5 mL methanol and 0.05 mL of ferrous chloride (2 mM). Then, 0.1 mL of ferrozine solution (5 mM) was added and the reaction mixture was incubated for 10 min in the dark at room temperature. Afterward, the absorbance of the samples was read at 562 nm by spectrophotometer. The EDTA was used as the reference standard. The results are expressed as the mean inhibition of the ferrozine–Fe^2+^ complex formation (%) ± SD and IC_50_ ± SD.

### 4.8 Statistical Analysis

The results of TPC determination and antioxidant assays were obtained from the average of three independent experiments. The data were analyzed by one‐way analysis of variance (ANOVA), followed by Duncan's multiple range test (DMRT), using the software SPSS v.21. *p* < 0.05 were considered statistically significant.

## Author Contributions

**Soukaina Abou‐Wakil**: Conceptualization, Investigation, Data curation, Writing – original draft. **Francesco Cacciola**: Conceptualization, Investigation, Data curation, Supervision, Writing – original draft, Writing – review and editing. **Maria Fernanda Taviano**: Conceptualization, Investigation, Data curation, Supervision, Writing – original draft, Writing – review and editing. **Mohamed Rochd**: Investigation, Data curation. **Roberto Laganà Vinci**: Investigation, Writing – original draft. **Federica Davì**: Investigation. **Fatima Housti**: Data curation,, Writing – review and editing. **Luigi Mondello**: Data curation, Writing – review and editing. **Nicola Micale**: Data curation, Writing – review and editing. **Natalizia Miceli**: Data curation, Writing – review and editing. All authors have read and agreed to the published version of the manuscript.

## Conflicts of Interest

The authors declare no conflicts of interest.

## Data Availability

The authors have nothing to report.
